# The burden of anxiety, depression, and stress, along with the prevalence of symptoms of PTSD, and perceptions of the drivers of psychological harms, as perceived by doctors and nurses working in ICUs in Nepal during the COVID-19 pandemic; a mixed method evaluation

**DOI:** 10.1186/s12913-024-10724-7

**Published:** 2024-04-10

**Authors:** Shirish KC, Tiffany E. Gooden, Diptesh Aryal, Kanchan Koirala, Subekshya Luitel, Rashan Haniffa, Abi Beane, Collaboration for Research, Implementation, and Training in Critical Care in Asia and Africa (CCAA), Diptesh Aryal, Shirish KC, Kanchan Koirala, Subekshya Luitel, Rohini Nepal, Sushil Khanal, Hem R Paneru, Subha K Shreshta, Sanjay Lakhey, Samina Amatya, Kaveri Thapa, Radhika Maharjan, Roshani Kafle, Anita Bashyal, Reema Shrestha, Dipika Khadka, Nilu Dullewe

**Affiliations:** 1 Nepal Intensive Care Research Foundation, Kathmandu, Nepal; 2 Institute of Applied Health Research, University of Birmingham, Birmingham, UK; 3 Centre for Inflammation Research, University of Edinburgh, Edinburgh, UK; 4 Mahidol Oxford Tropical Medicine Research Unit, Bangkok, Thailand

**Keywords:** Pandemic preparedness, psychological distress, COVID-19, Healthcare professionals, ICU, Critical care

## Abstract

**Background:**

The COVID-19 pandemic resulted in significant physical and psychological impacts for survivors, and for the healthcare professionals caring for patients. Nurses and doctors in critical care faced longer working hours, increased burden of patients, and limited resources, all in the context of personal social isolation and uncertainties regarding cross-infection. We evaluated the burden of anxiety, depression, stress, post-traumatic stress disorder (PTSD), and alcohol dependence among doctors and nurses working in intensive care units (ICUs) in Nepal and explored the individual and social drivers for these impacts.

**Methods:**

We conducted a mixed-methods study in Nepal, using an online survey to assess psychological well-being and semi-structured interviews to explore perceptions as to the drivers of anxiety, stress, and depression. Participants were recruited from existing national critical care professional organisations in Nepal and using a snowball technique. The online survey comprised of validated assessment tools for anxiety, depression, stress, PTSD, and alcohol dependence; all tools were analysed using published guidelines. Interviews were analysed using rapid appraisal techniques, and themes regarding the drivers for psychological distress were explored.

**Results:**

134 respondents (113 nurses, 21 doctors) completed the online survey. Twenty-eight (21%) participants experienced moderate to severe symptoms of depression; 67 (50%) experienced moderate or severe symptoms of anxiety; 114 (85%) had scores indicative of moderate to high levels of stress; 46 out of 100 reported symptoms of PTSD. Compared to doctors, nurses experienced more severe symptoms of depression, anxiety, and PTSD, whereas doctors experienced higher levels of stress than nurses. Most (95%) participants had scores indicative of low risk of alcohol dependence. Twenty participants were followed up in interviews. Social stigmatism, physical and emotional safety, enforced role change and the absence of organisational support were perceived drivers for poor psychological well-being.

**Conclusion:**

Nurses and doctors working in ICU during the COVID-19 pandemic sustained psychological impacts, manifesting as stress, anxiety, and for some, symptoms of PTSD. Nurses were more vulnerable. Individual characteristics and professional inequalities in healthcare may be potential modifiable factors for policy makers seeking to mitigate risks for healthcare providers.

**Supplementary Information:**

The online version contains supplementary material available at 10.1186/s12913-024-10724-7.

## Introduction

Between January 2020 and December 2021, the COVID-19 pandemic led to an estimated 18.2 million deaths [1]. Globally, healthcare systems were overwhelmed during the pandemic, with intensive care units (ICUs) receiving an unprecedented burden of patients [2]. In Nepal, the government first declared a lockdown on March 24, 2020, that lasted until July 21, 2020, and the second lockdown was announced on April 29, 2021, which was fully lifted on September 1, 2021 [3]. The first wave of the COVID-19 pandemic reached a peak of over 5000 cases a day in October 2020, and the second wave reached a peak of more than 9000 cases a day in May 2021, which was almost double [4]. Prior to the pandemic, Nepal reported a capacity of 1595 ICU beds across 194 hospitals and around 840 ventilators, equating to 2.8 ventilator-equipped ICU beds per 100,000 people [5]. To cope with the influx of COVID-19 patients, several existing postoperative wards and other high-dependency units of the hospitals were converted into improvised critical care units [6]. Globally, healthcare professionals (HCPs) and specifically those working in ICU and critical care services, arguably were at the frontline of the healthcare response. These HCPs faced the uncertainty of managing this new condition, extended working hours, limited personal protective equipment (PPE), and an increased risk of infection as they provide essential lifesaving interventions, including intubation and non-invasive respiratory management [7, 8].

The impacts of the COVID-19 pandemic on the mental health and well-being of HCPs who worked during and after this global emergency are slowly becoming apparent. Research emerging from China, the USA, and Europe [9] describes a significant burden of psychological distress and symptoms synonymous with mental health conditions in HCPs. This is also evident from the limited studies that have been conducted in Nepal. For instance, one study conducted among 150 HCPs from outpatient clinics and inpatient wards caring for COVID-19 patients in Nepal reported that 38% of participants suffered from anxiety and/or depression [10]. Another Nepali study revealed that the prevalence of anxiety and depression among HCPs, including health assistants and support staff was 47% and 41%, respectively [11]. A larger online survey of 475 HCPs including pharmacists, paramedics and public health practitioners reported similar findings (42% had anxiety) and noted that nurses had a higher proportion of symptoms compared to other HCPs [12].. Whilst these studies, in conjunction with a meta-analysis, indicate that depression, anxiety, and post-traumatic disorder (PTSD) are highly prevalent among HCPs during the pandemic [9–13], fewer studies have explored the disparities between professionals’ roles, specifically among ICU workers, a group exposed to more advanced cases of COVID-19. Indeed a small study in Nepal comprising 96 nurses revealed that nurses who worked directly with COVID-19 patients experienced more severe symptoms of depression and anxiety [13]. The nature and characteristics of mental health symptoms appear to vary geographically, the HCPs’ role, their individual characteristics (age, gender) along with health system’s pre-existing resource capacity and ability to respond to increasing demand placed by events such as a pandemic. Understanding the mental health impact of ICU workers, any disparities between professional roles and drivers behind poor mental health in Nepal will help to identify what support is needed for ICU workers for pandemic preparedness; thus, providing important directions for investment in health systems strengthening.

We aimed to investigate the burden of anxiety, depression, stress, PTSD, and alcohol dependence among doctors and nurses in Nepal that worked in the ICU during the COVID-19 pandemic. We further sought to identify the factors driving the self-reported burden of psychological distress by exploring the lived experiences of these two different professional groups, and how these experiences impacted their psychological health and well-being.

## Methods

### Study design

We undertook a mixed-methods cross-sectional study [14] in Nepal with ICU doctors and nurses, combining an online questionnaire consisting of validated self-assessment tools combined with semi-structured interviews. The following self-reporting psychological assessment tools were used, given they have been used in previous studies in other settings and their widely validated in a variety of settings: Beck Anxiety Inventory (BAI) [15], Beck Depression Inventory (BDI) [16], Perceived Stress Scale (PSS) [17], PTSD Checklist for Diagnostic and Statistical Manual of Mental Disorders-5 (PCL-5) [18] and Alcohol Use Disorder identification Tool (AUDIT) [19]. BDI, BAI, and AUDIT have been validated in Nepal [20–22] and the PSS has been tested for reliability and correlation in Nepal [23]. Whilst the PCL-5 has not been validated in a Nepali setting, it was piloted (along with all other assessment tools used) with 20 people before the study commenced. Participants were given the flexibility to complete the questionnaire in either Nepali or English language. Despite this option, all participants opted to respond in English.

#### Ethics approval

was granted from the Nepal Health Research Council (approval number: 176/2021 P). All participants provided informed consent electronically before completing the online questionnaire. Participants from the qualitative component provided further informed verbal consent before the interview commenced.

### Setting

In 2020, Nepal reported a capacity of 1595 ICU beds across 194 hospitals and around 840 ventilators, equating to 2.8 ventilator-equipped ICU beds per 100,000 people [5]. A year later, Nepal was under a state of health emergency, with patients being turned down due to a lack of ICU beds, oxygen, and ventilators [24].

### Participants and recruitment

Doctors and nurses with experience in caring for COVID-19 patients in Nepalese ICUs were eligible for participation. Initially doctors registered with the Nepalese Society of Critical Care Medicine (NSCCM) [25] and nurses registered with the Critical Care Nurses Association of Nepal (CCNAN) [26] were contacted and invited to participate. Both organisations consist of voluntary memberships and represent the doctors and nurses working in a critical care setting in Nepal. At the time of recruitment, there were 187 doctors and 104 nurses registered at these organisations. This initial purposive sampling was augmented by snowballing techniques, whereby respondents were invited to forward the questionnaire link to other doctors or nurses working in ICUs [27]. Following completion of the questionnaire, respondents were invited to participate in a virtual interview. A convenience sample of 20 participants (a number which, based on the literature, was likely to provide saturation of findings [28]) was subsequently scheduled for an interview.

### Study materials and data collection

The questionnaire was developed using an online survey platform (Google Forms) [29]. The questionnaire was piloted for readability and responder reliability with twenty HCPs based in Nepal, prior to roll out, who did not participate in the final analysis. Questionnaire content included socio-demographic information; age, sex, professional role and experience, degree of schooling, and home living arrangements; factors which had been identified as being important in the burden of psychological distress and impact on family life in similar research conducted during the previous SARS pandemic as well as the current COVID-19 event [30]. Participants could opt out of the study at any time. Participants could only complete the questionnaire once, and all survey responses were anonymous. Participants were signposted to healthcare services available to them should they be suffering from any distressing, mild, moderate or severe mental health symptoms. Invitations to participate in the questionnaire were sent out from 20th May 2021, and the questionnaire was closed to responses on 2nd October 2021.

The semi-structured interview topic guide was co-developed between doctors and nurses working in ICUs in Kathmandu. Co-design was used to ensure the sensitivity and appropriateness of the questions. None of the doctors and nurses involved in the codesign of the topic guide participated in the study proper. The qualitative component was aimed to augment the quantitative findings by providing an understanding of what social, organisational, and environmental factors were related to HCPs’ mental health. Topic guide questions focused on HCPs’ perceptions of their experiences of working during the pandemic and explored social, organisational, and environmental factors that may have influenced their self-reported burden and symptoms of psychological distress. These factors were selected from a review of the findings of the previously published meta-analysis and other studies conducted in Nepal [9–13]. The interview questions were piloted with five HCPs for interpretability and interviewer consistency. All interviews were conducted via video conferencing (Zoom) [31] between September 2021 and March 2022. Five ICU nurses with experience in conducting interviews and mixed methods research led the data collection following training on the topic guide. To ensure there was no prior relationship between the interviewer and the participant, interviewers were assigned to participants that worked in different ICUs than themselves and were not known to the interviewee. No one other than the interviewer and the participant was present for each interview, and interviews were conducted at the time chosen by the interviewee. Rapid assessment procedure (RAP) sheets were used for note-taking during the interviews [32]. Commonly used in rapid evaluations - designed to improve the rapidity and replicability of research during public health emergencies - RAP sheets help reduce the need for long-form transcription and encourage reflexivity for both interviewers and researchers, reduce interviewer bias, and enable validation of internal consistency with coding [33]. The RAP sheet contained the summary of questions from the topic guide, and the interviewers took notes of what the participants said regarding each question during the interview.

### Data analysis

Descriptive statistics were used to describe participants’ demographics and professional profiles. Psychological health and well-being assessment tools from the questionnaire were analysed using published guidelines. For the BDI, each of the 21 items corresponding to a symptom of depression was summed for each participant to give a single total score [16]. With each item ranging from 0 to 3 points, a total score of 13 or less was considered minimal to no depression, 14 to 19 as mild depression, 20 to 28 as moderate depression, and 29 to 63 as severe depression [16]. Data is also presented separately for suicidality (question 9 from the BDI) whereby anyone that said they have thoughts about or plans to kill themselves is said to have experienced suicidality. The BAI scores reported included the 21 symptoms of anxiety that ranged between 0 and 63 points [15]. The values for each symptom were summed, and a total score of 0 to 7 was interpreted as a minimal level of anxiety, 8 to 15 as mild, 16 to 25 as moderate, and 26 to 63 as severe anxiety [15]. Scores on the PSS ranged from 0 to 40, with higher scores indicating higher perceptions of stress [17]: scores ranging from 0 to 13 were considered low descriptors of stress; 14 to 26 moderate; and 27 to 40 were considered higher levels of perceived stress. For alcohol use disorder reported using AUDIT [19], a score of 0 indicated no previous or current alcohol use; a score of 1 to 7 suggested low-risk consumption; 8 to 14 hazardous or harmful alcohol consumption; 15 or higher indicated the likelihood of alcohol dependence (moderate to severe alcohol use disorder). The PCL-5 included 20 items with a score range of 0 to 80 and a score of 33 or higher, indicating the presence of PTSD [18]. A sensitivity analysis was conducted for the BDI, BAI and AUDIT scores based on local validation studies whereby a score of 15 or lower from the BDI indicated no depression [20], 12 or lower from the BAI indicated no anxiety [21], and a score of 11 or above from the AUDIT indicated discriminate dependent drinkers [22].

RAP sheets, along with interviewer notes, were reviewed by the research team before analysis to ensure information was complete. SK, KK and AB used a constant comparative method, coding data following each round of interviews and then reflecting back on the summary of the codes together with the interviewers to promote the accuracy of findings and reduce recall and interviewer bias. In addition, emerging themes identified following each round of coding were used to guide subsequent interviews [34]. The broader research team met following each coding round to review the findings and reflexivity [35]. Categories and the subsequent themes (‘drivers’) were developed through the iterative process of interviewing, coding, analysing, and reviewing.

## Results

We invited 120 doctors and 341 nurses to participate. A total of 21 doctors and 113 nurses responded, all of which completed the BDI, BAI, PSS, and AUDIT questions; 100 completed the PCL-5 (16 doctors and 84 nurses). Nearly all nurses were female (99%, *n* = 112), whereas most doctors were male (81%, *n* = 17). The characteristics of respondents are described in Table 1.

**Table 1:** Sociodemographic characteristics by profession and in total

	Doctors *n* = 21	Nurses *n* = 113	Total *n* = 134
Age, in years			
18–24	0 (0)	44 (38.9)	44 (32.8)
25–35	13 (61.9)	66 (58.4)	79 (59.0)
36 or above	8 (38.1)	3 (2.7)	11 (8.2)
Sex			
Male	17 (81.0)	0 (0)	17 (12.7)
Female	4 (19.0)	112 (99.1)	116 (86.6)
Prefer not to say	0 (0)	1 (0.8)	1 (0.8)
Education			
Proficiency certificate level	0 (0)	39 (34.5)	39 (29.1)
Bachelor’s degree	8 (38.1)	69 (61.1)	77 (57.5)
Master’s degree	10 (47.6)	5 (4.4)	15 (11.2)
PhD or fellowship	3 (14.3)	0 (0)	3 (2.2)
Work experience			
< 12 months	2 (9.5)	34 (30.1)	36 (26.9)
12 to 24 months	3 (14.3)	9 (8.0)	12 (9.0)
> 24 months	16 (76.2)	70 (61.9)	86 (64.2)
Marital status			
Married	11 (52.4)	35 (31.0)	46 (34.3)
Single	10 (47.6)	78 (69.0)	88 (65.7)
Living with a child (≤ 18 years), yes	11 (52.4)	22 (19.5)	33 (24.6)
Living with someone elderly (≥ 60 years), yes	13 (61.9)	48 (42.5)	61 (45.5)

50% (*n* = 67) of respondents reported experiencing symptoms associated with moderate to severe anxiety, and a further 27% (*n* = 36) scored for mild anxiety as a result of working in the ICU during the COVID-19 pandemic (Table 2). Anxiety levels (and associated symptoms) were more pronounced in nurses than doctors, with 55% (*n* = 62) of the former scoring moderate to severe on the anxiety scale, compared to 24% (*n* = 6) of the latter. 21% (*n* = 28) of respondents described symptoms associated with moderate to severe depression, with a near-even split between nurses and doctors. Three-quarters of respondents (*n* = 114; 85%) had scores indicative of moderate to high levels of stress; this proportion was higher among doctors (*n* = 19; 91%) compared to nurses (*n* = 95; 84%). Of the 100 individuals that completed the PCL-5 assessment (16 doctors and 84 nurses), 45% (*n* = 46) reported a constellation of symptoms closely associated with PTSD, with a higher prevalence among nurses (*n* = 40; 47%) compared to doctors (*n* = 6; 38%).

Using cut-off scores from Nepali validation studies, 45 (34%) participants were experiencing mild, moderate or severe depressive symptoms, 80 (60%) were experiencing mild, moderate or severe anxiety symptoms, and 3 (2%) were considered discriminate dependent drinkers. These results are in line with our main analysis, including that a greater proportion of nurses were still found to suffer from depression and anxiety symptoms (supplementary Table 1).

**Table 2:** Prevalence of symptoms as experienced by doctors and nurses

	Severity	Doctor, *n* = 21	Nurse, *n* = 113	Total, *n* = 134
Anxiety, BAI *N* (%)	Minimal	8 (38.1)	23 (20.4)	31(23.1)
	Mild	8 (38.1)	28 (24.8)	36 (26.9)
	Moderate	4 (19.1)	32 (28.3)	36 (26.9)
	Severe	1 (4.8)	30 (26.6)	31 (23.1)
Depression, BDI *N* (%)	Minimal	16 (76.2)	66 (58.4)	82 (61.2)
	Mild	1 (4.8)	23 (20.4)	24 (17.9)
	Moderate	2 (9.5)	17 (15.0)	19 (14.2)
	Severe	2 (9.5)	7 (6.2)	9 (6.7)
	Experienced suicidality	3 (14.3)	9 (8)	12 (8.9)
Stress, PSS *N* (%)	Low	2 (9.5)	18 (15.9)	20 (14.9)
	Moderate	17 (81.0)	82 (72.6)	99 (73.9)
	High	2 (9.5)	13 (11.5)	15 (11.2)
Alcohol dependence, AUDIT *N* (%)	Low risk / no consumption	18 (85.7)	110 (97.4)	128 (95.5)
	High risk	2 (9.5)	3 (2.7)	5 (3.7)
	Alcohol dependence	1 (4.8)	0 (0)	1 (0.8)
PTSD, PCL-5 *N* (%)		Doctor, *n* = 16	Nurse, *n* = 84	Total, *n* = 100
	Unlikely	10 (62.5)	44 (52.4)	54 (54.0)
	Probable	6 (37.5)	40 (47.1)	46 (45.0)

ICU: intensive care units; BAI: Beck Anxiety Inventory; BDI: Beck Depression Inventory; PSS: Perceived Stress Scale; AUDIT: Alcohol Use Disorder Identification Tool; PTSD: post-traumatic stress disorder; PCL-5: PTSD Checklist for Diagnostic and Statistical Manual of Mental Disorders-5

**Fig. 1:**
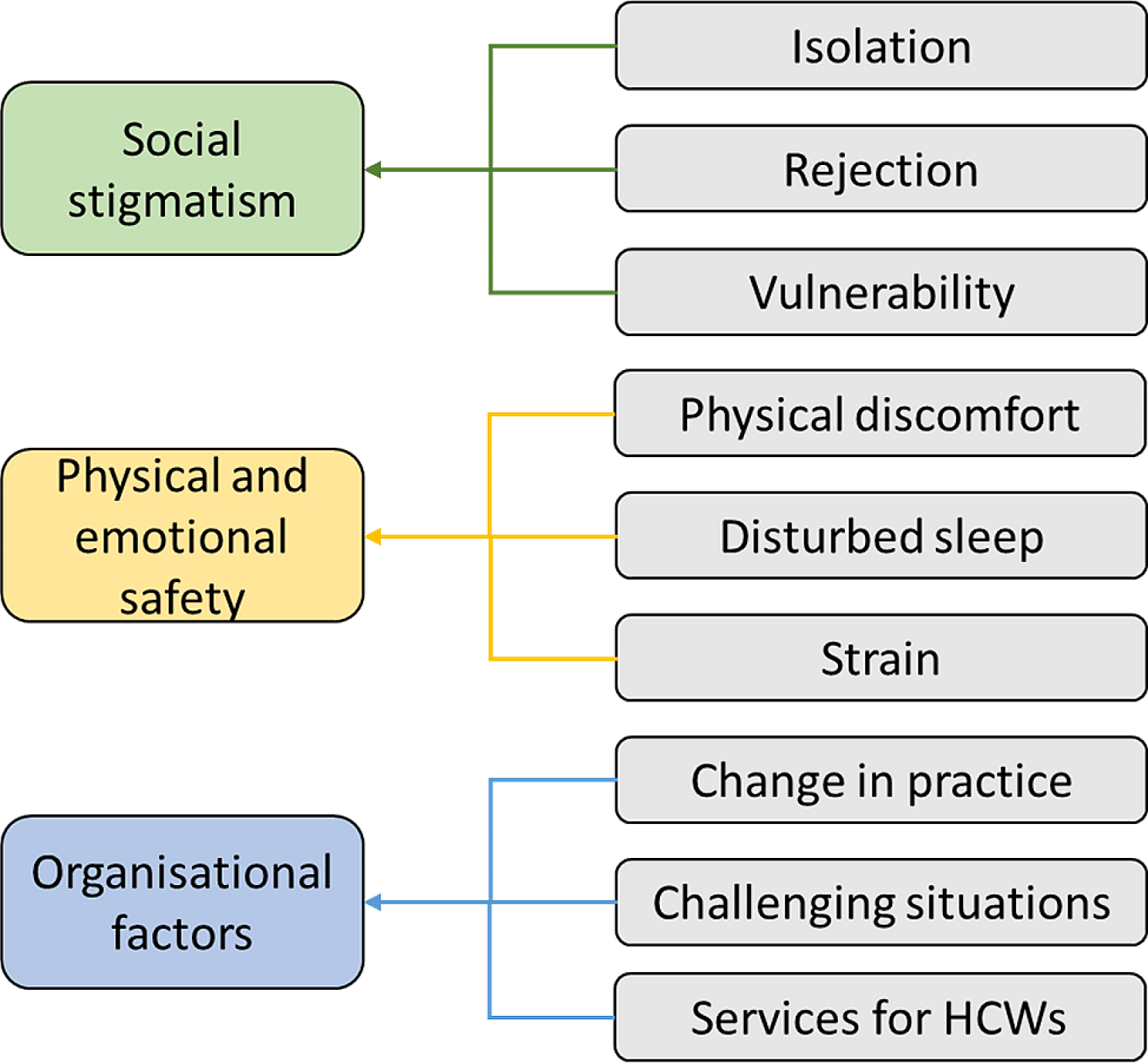
Coding tree for the four main drivers for psychological distress

Forty-six respondents to the online questionnaire volunteered to participate in the subsequent semi-structured interviews. Twenty participants were approached and consented to an interview: 16 were nurses (all female), and 4 were doctors (1 female, 3 male). On average, each interview resulted in 45 to 60 min of qualitative data. Saturation was met within the first 15 interviews, and findings were consistent between the coders and the research team. Analysis and synthesis of the interviews revealed nine themes, which, when codified, can be described as three key drivers of the psychological symptoms and impacts on mental well-being experienced by the interviewees: social stigmatism, physical and emotional safety, and organisational support. (Fig. 1). During the interviews, HCPs further described some of the coping strategies that they found helpful in mitigating the impacts experienced and may provide insights for future pandemic preparedness. These three themes, the drivers, and coping strategies, are explored below, along with quotes from the respondents.

### Social stigmatism

Interviewees described experiencing feelings of social stigmatisation as a result of interactions with their families, peers, as well as from the wider public. Examples of stigmatism experienced included physical avoidance from neighbours and community members when the HCP travelled to and from and around their home, especially when dwellings were in shared buildings and common areas.

“My house owner avoided talking and meeting me because I worked with COVID patients.” [N].

“I have an elderly family member, and I was afraid and worried [for them] when I came back from duty.” [N].

Interviewees described how rumours would spread within the community, notably related to concerns of risk of co-infection or cross-infection, either directly from parent to child or indirectly via friends and extended family. Some HCPs were asked or elected to stay away from their home so as to reduce the stigma to them and their family and in an attempt to reduce the risk of co-infection, particularly when they had vulnerable family members. Interviewees described how this self-selected or enforced separation and isolation resulted in feelings of rejection, physically and emotionally heightened feelings of stress and anxiety, alongside the threat to physical and emotional safety.

“My house owner avoided talking and meeting me because I worked with COVID patients.” [N].

“I have an elderly family member, and I was afraid and worried [for them] when I came back from duty.” [N].

### Physical and emotional safety

Increased workload and an enforced change in working pattern/ shift structures were experienced by all the HCPs interviewed. These longer overall working hours, increased duration of shift patterns, and enforced working rotas were perceived as resulting in a loss of physical and emotional safety by the interviewees. Feelings of loss of control, insomnia, or disruption to sleep patterns, alongside physical discomfort through sustained working in personal protective equipment, often in hot and humid temperatures. This physical and mental endurance contributed to feelings of emotional stress and anxiety.

“Shift frequency was increased, and I only got one night off in a week. Sometimes I had to work extra hours, which was very stressful.” [N].

“My sleep pattern had changed, I felt restless and was afraid about COVID” [D].

The change in shift structure and in working patterns meant for some HCPs enforced separation from family and friends whereby HCPs sought accommodation away from family or in temporary lodgings. This again resulted in isolation and additional strain on other family members so as to provide care for HCP’s dependents.

“I had to involve other family members to arrange for the medication and care of my grandmother” [N].

Increased working hours and changes in working patterns further had physical impacts; participants described skipping meals or having limited time to eat. The need to wear personal protective equipment (PPE), and indeed the risks to safety when PPE was not available, associated risks of non-availability of equipment, brought with it a risk to physical and emotional safety. HCPs interviewed reported skin lacerations, irritation, and discomfort whilst wearing equipment in hot, humid working environments.

“We had to frequently change the PPE and masks, which has caused skin problems that still exist.” [N].

### Organisational support

Interviewees found the COVID-19 pandemic brought new and often enforced work responsibilities, some of which were associated with high levels of professional anxiety, stress, and uncertainty. A professionally challenging situation, even for those with many years of ICU working experience. HCPs faced emotionally challenging tasks such as dealing with end-of-life situations (particularly without relatives of the patient present) and having to comfort relatives over the phone, of which they received limited to no training or support on handling such situations.

“I went through an emotional breakdown while dealing with the end of the life situation of patients without the presence of family members in the COVID ICU… I felt sad when a young patient lost their lives” [D].

“Accommodation or isolation facilities should be provided by the hospital” [D].

“If incentives were provided in time and staff were provided with health insurance it would motivate us” [N].

Ever-changing role and responsibilities created anxiety for HCPs as to what care to deliver, and the rapidity and uncertainty of care were associated with feelings of vulnerability. Interviewees expressed how they wished there was a need for greater organisational support to better cope with the frequent updates and changes to practice. Furthermore, HCPs expressed concerns regarding a shortage of staff and the lack of mental health counselling and support, accommodation on-site at the hospital, and transportation to and from work.

“Mental health support or counselling facilities were not provided. It should be there… seniors and hospital staff should also talk to the staff to know the situation.” [N].

“Safety of healthcare workers should be the priority and nurse-patient ratio should be maintained to provide quality care to the patients… hospital should have recruited more staff.” [N].

### Coping strategies

Participants described various ways in which they coped with the emotional, physical, social, and professional impacts of working through the pandemic. This included speaking with family and friends about the pressures they were under, taking up activities in their off time, such as gardening and reading, and using media entertainment such as music, movies, and shows. A few participants also mentioned that comparing the situation in Nepal to other countries (i.e., keeping up-to-date with the news) also helped them cope. Others mentioned that detachment from social media and more self-awareness through meditation helped.

“I ventilated my feelings with friends and family. Listening to soothing music also helped me cope with the stress.” [N].

“I coped by gardening with my sister in my home.” [N].

“I… watched the news that compared the death rates, which was low compared to others.” [D].

## Discussion

The COVID-19 pandemic’s impact on healthcare services and population health internationally is unprecedented in recent times. As healthcare professionals, policymakers, and researchers work to strengthen services in preparation for future pandemics now and mitigate the long-term impacts on individual and population health, understanding the impact on and perspectives of doctors and nurses at the frontline of care can provide important learning regarding the individuals characteristics and professional, social and economic drivers which may increase the risk of psychological impacts.

Mandated and enforced changes in role, specifically in working hours and shift patterns, were a key driver of psychological anxiety and distress. Within hospitals in Nepal, many departments were closed, and stay-at-home orders meant that outpatient or clinical services all but ceased. This resulted in an increased role and scope for critical care trained staff, and in contrast to other health systems (such as the UK) where healthcare staff were redeployed to ICU, there was a separation for ICU staff even from their professional peers working in other specialties. The increased scope and uncertainty of the HCP’s role, along with limited choice in redeployment in the ICU was another driver of poor mental health- and dominated nursing participants’ experiences. Interviewees described how these changes impacted not only themselves but the multigenerational families for whom many cared for. This enforcement of role change, and the related descriptions of the drivers for these impacts as experienced by participants in this study point not only to the differences in roles between nurses and doctors; but also highlights disparities in autonomy, advocacy for role change during international emergencies, and the implications of work on home and family life [36].

Giving staff choice to select shift patterns and ensuring the opportunity to have periods of rest to reconnect with family and have self-care is needed. Consultation and shared decision-making, even in times of restricted choice, are associated with improved perceptions of work from staff and may result in reducing psychological distress and promoting emotional safety, which is, in turn, associated with better outcomes for patients [37, 38]. However, nurses in Nepal, as with many health systems, may have less opportunity for strategic and organisational decision making in response to public health emergencies. The impact of ongoing disparities between professionals and their agency to advocate for wellbeing and safety warrants further research.

Nurses were disproportionately burdened by both occurrence and severity of symptoms of anxiety and depression as a result of their work during the pandemic when compared to doctors.

Nearly half of all respondents had symptoms of anxiety and PTSD (again more prevalent in nurses), and the burden of anxiety symptoms was higher than the reported 22–33% from a recent umbrella review [39]. The burden of stress we report was also higher than a smaller study conducted in Nepal during the pandemic, which reported stress among 53.2% of healthcare professionals working in hospitals, primary health centres, pharmacies, and health posts in Nepal [40]; it was also higher than a meta-analysis of published studies exploring the incidence of both stress (57%) and PTSD (22%) among all cadres of healthcare workers [41]. One reason for the higher reported symptoms in our study may be the focus on ICU workers and their role in the management of end-of-life care. Indeed, our results for depression and anxiety are comparable to a study involving nurses working directly with COVID-19 in Nepal [13]. Studies conducted elsewhere in Asia have highlighted this positive relationship between ICU experiences and poor mental health [42].

Nurses in Nepal, as with many other countries, are more likely to be female, younger in age, and have less opportunity for graduate study; and have lower earning potential than physician colleagues [43]; all characteristics associated with increased risk of poorer mental health outcomes [44]. Exploration into the disparities of the psychological and health impacts of COVID-19 on different cadres of healthcare workers is emerging. A systematic review conducted in 2020, identified 27 studies which sought to explore the disparity in impacts of the pandemic on HCP’s psychological well-being. The findings from the review are in line with ours, indicating that the burden of symptoms for anxiety, depression, and PTSD is higher in nurses compared to doctors [45]. Notably only a few of these studies used validated tools for assessment of specific symptoms of anxiety, depression, or substance misuse [45]. Our study serves to strengthen the evidence of the vulnerability of nurses.

Nepal, like many other lower and middle-income countries in South and Southeast Asia, enforced large-scale lockdowns and restrictions of movement for all but essential healthcare and municipal staff [46]. As such, social stigmatism, physical and emotional safety, and organisational support were key drivers behind the elevated symptoms of psychological distress in ICU HCPs and may be a key determinant of differences between health systems internationally. Furthermore, the family responsibilities and social circumstances for nurses, contributed to their experiences of isolation, rejection, vulnerability, physical discomfort, and strain. These drivers mirrored those reported from Europe; and may reflect differences experienced by nurses as a result of their gender, and role norms of primary family carers within society [44].

Interviewees from both professional groups expressed concern at the absence of preparedness and support they felt from their employing institutions. This is notable given the ongoing investment in pandemic preparedness and the potential to make changes now to prepare for the next pandemic or public health emergency. Interventions such as resilience training, scenario-based simulation training, and group exercises based on psychoeducation and cognitive behavioural therapy (CBT) principles have proved effective in reducing anxiety, depression, stress, and PTSD among doctors and nurses while simultaneously improving their ability to work in unprecedented situations in other sectors [47]. Similar provisions may be valuable for ICU-based healthcare professionals and are deliverable online, making rollout potentially more feasible.

### Strengths and limitations

A strength of this study is the exploration of participants’ perspectives on the drivers behind the burden of poor mental health described in ICU HCPs. This mixed methods approach offers insights into doctors’ and nurses’ unique individual, social and professional characteristics that may be associated with increased risk of distress. These differences and their potential for disparity in impacts on health and wellbeing should be of interest to policymakers and healthcare facility managers involved in future pandemic preparedness. However, the study has some limitations to acknowledge. Given the use of the snowball technique, we were able to ensure a high number of respondents, but as a consequence, we were unable to track the number of respondents that came from using this technique compared to those initially invited from the NSCCM and CCNAN. Therefore, a response rate and, subsequently, a non-response rate could not be reported. We did not collect information on the level of training in critical care that participants received; trained health professionals are likely to have additional skills in how to handle the potential stressful environment in critical care settings. Also, due to the lack of validation of the PCL-5 in Nepal, the results of this assessment tool should be interpreted with caution. The survey tools used for this study have not been validated in an online format. However, given these tools were self-reporting, and were piloted and administered in English, the online format is thought to have minimal impact on the results. Additionally, participants for the qualitative component were recruited based on convenience sampling; therefore, the diversity of the sample may not be optimised. We acknowledge that recall bias may be present in the participants during the interview, given they were recalling their experiences throughout the pandemic for up to 24 months prior to the interview; however, we hope the piloting of the interviews, the use of multiple researchers to code the data, and the constant comparative nature of the evaluation will mitigate this potential.

## Conclusion

The COVID-19 pandemic negatively impacted the mental health of HCPs worldwide. This study strengthens existing evidence that nurses were (and may remain) at increased risk of both cross infection and may also be more vulnerable to psychological impacts including anxiety, depression and PTSD than their professional colleagues. In addition, critical care staff may be at even greater risk, due to the uniqueness of their role which includes prolonged periods of time with infected patients, frontline role in managing end of life care, and as described here, limited ability to advocate for changing role and working patterns during an emergency. Professional hierarchies, and social-economic and gender profiles unique to nurses, may be potential drivers for these disparities, and warrants further research. Learning from the ICU HCPs’ experiences during the COVID-19 pandemic may inform future preparedness strategies e to mitigate short and long-term mental illness among ICU HCPs in future pandemics.

### Electronic supplementary material

Below is the link to the electronic supplementary material.

Supplementary Material 1

## Data Availability

The interview guide is available in the Figshare repository, 10.6084/m9.figshare.24247384.v1. The data supporting the conclusions of this article are available in the Figshare repository, 10.6084/m9.figshare.23999790.v1.

## Abbreviations

COVID-19: Coronavirus disease 2019
ICU: Intensive care unit
HCP: Healthcare professional
PPE: Personal protective equipment
PTSD: Post-traumatic stress disorder
NSCCM: Nepalese Society of Critical Care Medicine
CCNAN: Critical Care Nurses Association of Nepal
BAI: Beck Anxiety Inventory
BDI: Beck Depression Inventory
PSS: Perceived Stress Scale
PCL-5: PTSD Checklist for Diagnostic and Statistical Manual of Mental Disorders-5
AUDIT: Alcohol Use Disorder Identification Tool
RAP: Rapid assessment procedure
CBT: Cognitive behavioural therapy
