# Supplementary material for: The burden of anxiety, depression, and stress, along with the prevalence of symptoms of PTSD, and perceptions of the drivers of psychological harms, as perceived by doctors and nurses working in ICUs in Nepal during the COVID-19 pandemic; a mixed method evaluation
Source: BMC Health Serv Res. 2024 Apr 10;24:450. doi: 10.1186/s12913-024-10724-7 (PMC11007980; doi:10.1186/s12913-024-10724-7)
Supplement: Supplementary file 1 — Supplementary Material 1 [file 12913_2024_10724_MOESM1_ESM.docx]

|  | Doctors, N=21 | Nurses, N=113 | Total, N=134 |
| --- | --- | --- | --- |
| **BDI** |  |  |  |
| No depression  ≤ 15 | 17 (80.9) | 72 (63.7) | 89 (66.4) |
| Mild to severe depression  ≥ 16 | 4 (19.1) | 41 (36.3) | 45 (33.6) |
| **BAI** |  |  |  |
| No anxiety  ≤ 12 | 14 (66.7) | 40 (35.4) | 54 (40.3) |
| Mild to severe anxiety  ≥ 13 | 7 (33.3) | 73 (64.6) | 80 (59.7) |
| **AUDIT** |  |  |  |
| No harmful drinking  ≤ 10 | 19 (90.5) | 112 (99.1) | 131 (97.8) |
| Discriminate dependent drinkers  ≥ 11 | 2 (9.5) | 1 (0.9) | 3 (2.2) |

Supplementary table 1. Sensitivity Analysis Based on Nepali Validated Studies
